# Analysis of differentially expressed genes in human hepatocellular carcinoma using suppression subtractive hybridization

**DOI:** 10.1054/bjoc.2001.1901

**Published:** 2001-07

**Authors:** Y Miyasaka, N Enomoto, K Nagayama, N Izumi, F Marumo, M Watanabe, C Sato

**Affiliations:** Department of Gastroenterology and Hepatology, Second Department of Internal Medicine, Department of Health Science, Tokyo Medical and Dental University, Tokyo, Japan; Department of Gastroenterology and Hepatology, Musashino Red Cross Hospital, Tokyo, Japan

**Keywords:** suppression subtractive hybridization, hepatocellular carcinoma, focal adhesion kinase, pepsinogen C, decorin

## Abstract

The genetic basis of hepatocellular carcinoma (HCC) has not yet been fully understood. Although various methods have been developed to detect differentially expressed genes in malignant diseases, efficient analysis from clinical specimens is generally difficult to perform due to the requirement of a large amount of samples. In the present study, we analysed differentially expressed genes with a small amount of human HCC samples using suppression subtractive hybridization (SSH). Total RNA were obtained from the hepatitis C virus-associated HCC and adjacent non-HCC liver tissues. cDNA was synthesized using modified RT-PCR, and then tester cDNA was ligated with 2 different kinds of adaptors and hybridized with an excess amount of driver cDNA. Tester specific cDNA was obtained by suppression PCR and the final PCR product was subcloned and sequenced. We identified 7 known genes (focal adhesion kinase, deleted in colon cancer, guanine binding inhibitory protein α, glutamine synthetase, ornithine aminotransferase, M130, and pepsinogen C) and 2 previously unknown genes as being overexpressed in HCC, and 1 gene (decorin) as suppressed in HCC. Quantitative analysis of gene expression using quantitative RT-PCR demonstrated the differential expression of these genes in the original and other HCC samples. These findings demonstrated that it is possible to identify the previously unknown, differential gene expression from a small amount of clinical samples. Information about such alterations in gene expression could be useful for elucidating the genetic events in HCC pathogenesis, developing the new diagnosic markers, or determining novel therapeutic targets. © 2001 Cancer Research Campaign http://www.bjcancer.com


*British Journal of Cancer* (2001) **85**(2), 228–234
© 2001 Cancer Research Campaign
doi: 10.1054/ bjoc.2001.1901, available online at http://www.idealibrary.com on IDEAL®

### Analysis of differentially expressed genes in human hepatocellular carcinoma using suppression subtractive hybridization

**Y Miyasaka[1,2], N Enomoto[1,2], K Nagayama[1,2], N Izumi[4], F Marumo[2], M Watanabe[1] and C Sato[3]**

[1]Department of Gastroenterology and Hepatology and [2]Second Department of Internal Medicine; [3]Department of Health Science, Tokyo Medical and Dental University, Tokyo, Japan; [4]Department of Gastroenterology and Hepatology, Musashino Red Cross Hospital, Tokyo, Japan

**Summary** The genetic basis of hepatocellular carcinoma (HCC) has not yet been fully understood. Although various methods have been developed to detect differentially expressed genes in malignant diseases, efficient analysis from clinical specimens is generally difficult to perform due to the requirement of a large amount of samples. In the present study, we analysed differentially expressed genes with a small amount of human HCC samples using suppression subtractive hybridization (SSH). Total RNA were obtained from the hepatitis C virus-associated HCC and adjacent non-HCC liver tissues. cDNA was synthesized using modified RT-PCR, and then tester cDNA was ligated with 2 different kinds of adaptors and hybridized with an excess amount of driver cDNA. Tester specific cDNA was obtained by suppression PCR and the final PCR product was subcloned and sequenced. We identified 7 known genes (focal adhesion kinase, deleted in colon cancer, guanine binding inhibitory protein α, glutamine synthetase, ornithine aminotransferase, M130, and pepsinogen C) and 2 previously unknown genes as being overexpressed in HCC, and 1 gene (decorin) as suppressed in HCC. Quantitative analysis of gene expression using quantitative RT-PCR demonstrated the differential expression of these genes in the original and other HCC samples. These findings demonstrated that it is possible to identify the previously unknown, differential gene expression from a small amount of clinical samples. Information about such alterations in gene expression could be useful for elucidating the genetic events in HCC pathogenesis, developing the new diagnosic markers, or determining novel therapeutic targets. © 2001 Cancer Research Campaign http://www.bjcancer.com

**Keywords**: suppression subtractive hybridization; hepatocellular carcinoma; focal adhesion kinase; pepsinogen C; decorin

Hepatocellular carcinoma (HCC) is one of the most common tumours in the world and its prognosis is poor (Sherlock and Dooley, 1997). However, the molecular mechanisms that lead to the development and progression of HCC remain unclear. Identification and characterization of specifically up- or down-regulated genes in human HCC in comparison with the surrounding non-tumorous tissue is useful for understanding molecular changes in HCC and for developing diagnostic markers and new potential therapeutic targets.

Several methods have been reported for detecting differentially expressed genes in tumorous tissues as compared with the corresponding non-tumorous tissues, such as differential display (DD) (Liang et al, 1992), expressed sequenced tags (EST) analysis (Vasmatzis et al, 1998), subtractive hybridization (el-Deiry et al, 1993) and serial analysis of gene expression (SAGE) (Velculescu et al, 1995). More recently, microarray technology attracted great interest and continues to hold promise for studies on human disease states (Khan et al, 1998; Alon et al, 1999). However, these methods are often laborious and generally require a large amount of mRNA, which is difficult to obtain from clinical materials, especially from small HCC specimens.

*Received 10 January 2001*
*Revised 23 April 2001*
*Accepted 30 April 2001*

*Correspondence to:* N Enomoto

In the present study, in order to screen differentially expressed genes efficiently from small clinical specimens of HCC, we utilized suppression subtractive hybridization (SSH) (Diatchenko et al, 1996; von Stein et al, 1997) combined with modified RT-PCR technology (Matz et al, 1999). Although several studies using SSH have successfully identified the differentially expressed genes in a variety of disease states (Kuang et al, 1998; Stubbs et al, 1999), the present study is the first report to identify the altered gene expression profiles in HCC by SSH.

### MATERIALS AND METHODS

### Tissue samples

We used an HCC sample (moderately differentiated hepatocellular carcinoma) and an adjacent non-HCC liver tissue sample, both obtained during surgery from a 56-year-old man infected with hepatitis C virus, for the initial SSH analysis. The HCC was 3 cm in diameter and the histology of non-HCC tissue was consistent with chronic hepatitis, with moderate inflammatory activity and severe fibrosis. The other 9 pairs of HCC and adjacent non-HCC tissues were obtained at surgery or needle biopsy. In all, 10 pairs of HCC and non-HCC samples were enrolled in this study, of which 8 cases were infected with hepatitis C virus, while the other 2 cases were due to hepatitis B virus and primary biliary cirrhosis. Among the 10 cases, 4 well-differentiated adenocarcinomas, 5 moderately differentiated adenocarcinomas, and one poorly differentiated adenocarcinoma were present. Written informed consent was obtained from each patient before liver biopsy or surgery, and the study protocol conformed to the ethical guidelines of the 1975 Declaration of Helsinki. In addition, institutional approval was obtained.

### RNA extraction and SMART™ cDNA synthesis

Total RNA was extracted by the modified acid-guanidium-chloroform method (Chomczynski et al, 1987) using ISOGEN™ (Nippon Gene, Toyama, Japan) and according to the manufacturer's instruction. We generated cDNA from the total RNA samples from 10 pairs of HCC and adjacent non-HCC samples using the SMART™ (Switch Mechanism at 5′ end of RNA Template) PCR cDNA synthesis kit (Clontech, Palo Alto, CA). The SMART™ cDNA synthesis technology (Matz et al, 1999) utilizes a combination of 2 primers in a single reaction. Briefly, 1 μg of total RNA was reverse-transcribed in 10 μl mixture with 200 U of Superscript™ reverse transcriptase (Gibco, Madison, WI) using 10 μM of modified oligo dT primer (CDS primer; 5′AAGCAGTGGTAACAACGCAGAGTACT30(AGC)(AGCT) and SMART™ primer (5′AAGCAGTGGTAACAACGCAGAG-TACGCGGG). The CDS primer is used to prime the first-strand reaction, while the SMART™ oligonucleotide serves as a short, extended template at the 5′ end of the RNA template. When the reverse transcriptase reaches the 5′ end of the mRNA, the enzyme's terminal transferase activity adds a few additional deoxycytidines to the 3′ end of the cDNA. The SMART™ oligonucleotide, which has an oligo (G) sequence at its 3′ end, base-pairs with the deoxycytidine stretch, creating an extended template. The enzyme switches templates and continues replicating to the end of the SMART™ oligonucleotide. The resulting full-length, single-stranded cDNA contains the complete 5′ end of the mRNA and the sequence complementary to the SMART™ oligonucleotide, which then serves as a long distance PCR priming site to amplify the full-length cDNA. The first-strand of cDNA was diluted to a final volume of 50 μl with 1 × TE buffer (10 mM Tris-HCl, pH 8.0, 1 mM EDTA). One μl of the diluted cDNA was used to generate the cDNA by long distance PCR with Advantage™ Klen Taq polymerase mix (Clontech, Palo Alto, CA), using PCR primer (5′ AAGCAGTGGTAACAACGCAGA) following the manufacturer's instructions.

### Suppression subtractive hybridization (SSH)

SSH was performed with the PCR-Select™ cDNA Subtraction Kit (Clontech, Palo Alto, CA) according to the manufacturer's protocol, except for slight modifications as below. SMART™ cDNA derived from HCC and non-HCC tissues were digested by RsaI restriction enzymes to obtain blunt-ends which are necessary for adaptor ligation. RsaI digested cDNA was purified using the QIA quick™ PCR Purification Kit (Qiagen, Chatsworth, CA). Tester cDNA was divided into two and ligated separately with 2 different adaptors, and cDNA without adaptors was used as a driver. 0.25 ng of tester cDNA with an excess amount (150 ng) of driver cDNA were hybridized in one μl of hybridization mixture at 65˚C for 16 hours. The 2 hybridization solutions and 150 ng of a fresh driver cDNA were then mixed and incubated at 65˚C for additional 8 hours, so that the remaining equalized single-stranded tester cDNA was hybridized with excess driver cDNA. Thus, only tester-specific cDNA formed the double-stranded cDNA with different adaptors on each end. They were selectively amplified by suppression PCR followed by the nested PCR, which does not exponentially amplify the non-adaptor (derived from driver cDNA), cDNA with the one adaptor on either end (derived from tester cDNA hybridized with driver cDNA), or cDNA with the same adaptor on both ends (derived from relatively abundant tester cDNA).

### Cloning and sequencing

10 ng of PCR products were cloned into plasmids pGEM-T Easy Vector™ (Promega, Madison, WI) and transformed to competent *E. coli* XL2-blue™ Ultracompetent cells (Stratagene, Ceder Creek, TX). 100 colonies were randomly picked up and sequenced using the PRISM dye termination kit™ (ABI, Chiba, Japan). BLAST Search 2.0 (www.ncbi.nlm.nih.gov/blast/blast.cgi) was used to analyse sequence homologies in the gene database. For sequences with no significant homology to the database, we performed 3′ and 5′-rapid amplification of cDNA ends (RACE) in order to obtain the full-length cDNAs of these transcripts using a Marathon cDNA Amplification kit™ (Clontech, Palo Alto, CA) according to the manufacturer's protocol.

### Immunohistochemistry

Immunohistochemical staining was performed in the index HCC and surrounding non-HCC tissue with 5 μm thick sections from formalin-fixed, paraffin embedded blocks, using the labelled streptavidin biotin immunohistochemical staining method, in order to confirm the differential expression of these genes in the original case. Sections were incubated with anti-pepsinogen C antibody (Biogenesis, Poole, UK), anti-GS antibody, anti-FAK antibody, and anti-DCC antibody (Santa Cruz Biotechnology, Inc, Santa Cruz, CA) at a dilution of 1:100 overnight at 4˚C. Then, tissue sections were exposed to biotin-labelled anti-mouse IgG (DAKO, Glostrup, Denmark) for reaction with pepsinogen C, and anti-goat IgG (DAKO, Glostrup, Denmark) for glutamine synthetase for 30 min at room temperature and then with streptavidin (DAKO, Glostrup, Denmark). Visualization was performed by using 3,3′-diaminobenzidine and $H_2O_2$ and counterstained with haematoxylin.

### Quantitative analysis of overexpressed genes

Overexpression of the obtained genes was confirmed by semi-quantitative RT-PCR, comparing the amount of PCR products by agarose gel electrophoresis at the PCR cycle number in the exponential phase of amplification as indicated in Figure 5. Subsequently, the mRNA expression levels of these genes were quantitated by competitive RT-PCR using gene-specific deleted cDNA competitors (Zachar et al, 1993) (focal adhesion kinase, deleted in colon cancer, glutamine synthetase, ornithine amino-transferase, guanine binding inhibitory protein, and M130) or by the real-time PCR (pepsinogen C and decorin) using the Light Cycler System™ (Roche Diagnostics, Manheim, Germany) (Wittwer et al, 1997). Expression of each mRNA, standardized with glyceroaldehyde-3-phospho-dehydrogenase (G3PDH) expression, was compared in 10 pairs of HCC and adjacent non-HCC tissues. The primers used in the quantitative PCR were shown in Table 1.

© 2001 Cancer Research Campaign 

**Table 1** Primer sequences

| Primer name | Sequence |
|---|---|
| FAK forward | 5′ TTCATTATTTTGAAAGCAATAGT3′ |
| FAK reverse | 5′ CAACCCAACTTCAAAGCAATTTC3′ |
| FAK forward competitor | 5′ TGCCTATTAAATGGATGGCTCCAATGGTGTGAAGCCTTTTCAA3′ |
| DCC forward | 5′ CGAGTTGTGGCTTACAATGAATGG3′ |
| DCC reverse | 5′ CCACTTCCAGGGAGACGTTCTGAG3′ |
| DCC forward competitor | 5′ CGAGTTGTGGCTTACAATGAATGGTGCAAGCTGTATCTACCTCA3′ |
| GS forward | 5′ GTACTCTGGTTAGGTTAGGACTT3′ |
| GS reverse | 5′ TTCTAATCCGACTATTTGTCTCA3′ |
| GS forward competitor | 5′ GTACTCTGGTTAGGTTAGGACTTGTAGGGGTTGGGAATCAGAG3′ |
| OAT forward | 5′ GGGAGCATGGGTCCACATACGGT3′ |
| OAT reverse | 5′ CACATCAAAACACTTCAACTGAA3′ |
| OAT forward competitor | 5′ GGGAGCATGGGTCCACATACGGTAAAATGCAGACAAATTGGGC3′ |
| Giα forward | 5′ GCCCTCTCACTATATGCTATCCA3′ |
| Giα reverse | 5′ ACTCATTTGGTTTGAAAATGCA3′ |
| Giα forward competitor | 5′ GCCCTCTCACTATATGCTATCCAGGACACAAAGGAAATATACA3′ |
| M130 forward | 5′ ACGCTGGGGCCATAGTGAGTGTG3′ |
| M130 reverse | 5′ ACAGACCTGAGGAATTCATTAGG3′ |
| M130 forward competitor | 5′ ACGCTGGGGCCATAGTGAGTGTGCTCATCCCGTCAGTCATCCT3′ |
| PGC forward | 5′ CAGCTTGACCTTCATCATCAATG3′ |
| PGC reverse | 5′ CCAGAGTGGAAAGACAGATACAA3′ |
| Decorin forward | 5′ AAATAACTGAAATCAAAGATGGAGA3′ |
| Decorin reverse | 5′ TAAGAGAAGGAGGAAGACCTTGAGG3′ |

### RESULTS

### SMART™ RT-PCR

From 20 ng of total RNA, 1–2 μg of cDNA were generated by the SMART™ RT-PCR protocol, yielding a smear of cDNA from 0.5 to 6 kb with many bright bands corresponding to relatively abundant transcripts on agarose gel electrophoresis (data not shown).

### SSH

In order to evaluate the efficiency of our SSH method, we created the artificial tester HCC cDNA in which bacteriophage ϕ χ 174/HaeIII DNA was added, so that each fragment of ϕ χ 174 DNA corresponded to about 0.02% (1/5000) of the HCC cDNA. Using the HCC cDNA by itself without ϕ χ 174 DNA as the driver, SSH was carried out. DNA fragments derived from ϕ χ 174 DNA were successfully amplified by SSH, indicating that our SSH method could detect the differentially expressed gene with as little as 1/5000 of tester cDNA (data not shown).

After SSH using HCC cDNA as a tester, there remained approximately 10 bands as a second PCR product on agarose gel electrophoresis (Figure 1). The nucleotide sequences of 100 clones obtained from this PCR product were analyzed by a BLAST 2.0 database homology search. Seven known genes, pepsinogen C (PGC, Gene Bank Accession Number, P20142), focal adhesion kinase (FAK, Q05397), glutamine synthetase (GS, P43146), M130 (VI38005), guanine binding inhibitory protein α (Giα, P08754), deleted in colon cancer (DCC, P43146), and ornithine aminotransferase (OAT, P04181) were repetitively detected in 100 randomly selected clones (Table 2). Two sequences (arbitrarily designated as HCC-1 and HCC-2), without significant homology to genes within the database, were also repeatedly isolated. The sequence analysis of RACE products from these clones revealed weak homologies to retrotransposon MARINER (U49974) and LINE-1 (B28096), respectively (Figure 2). Using non-HCC cDNA as a tester, decorin (P07585) was identified as suppressed in HCC.

### Confirmation of differentially expressed genes by semi-quantitative RT-PCR

Differential expression of genes detected in SSH analysis were confirmed in original HCC and non-HCC tissues by semi-quantitative RT-PCR as shown in Figure 3. PCR products in the exponential phase of amplification were analysed by agarose gel electrophoresis, which compared the amount of specific products

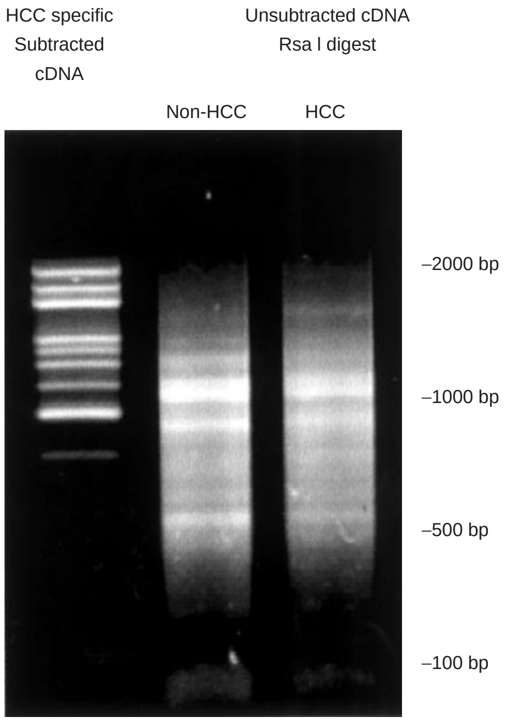

**Figure 1** SMART™ cDNA after Rsa I digestion of HCC and non-HCC cDNA, and nested PCR products of SSH on electrophoresis on 2.0% agarose/EtBr gel

 *© 2001 Cancer Research Campaign*

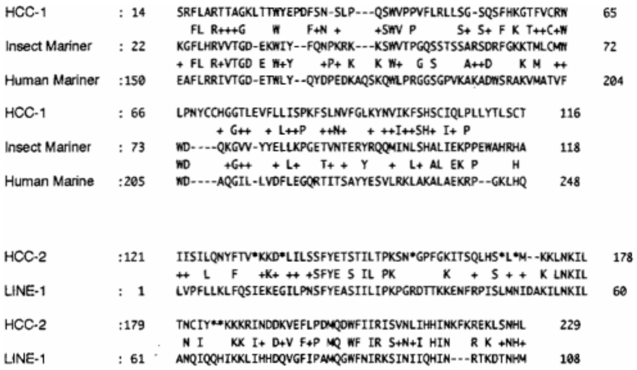

**Figure 2** Alignment of HCC-1 with human and insect MARINER transposase, and HCC-2 with human LINE-1. Identical amino acids are shown between the sequences. A (+) sign indicates a conservative replacement. Insect MARINER is the milkweed bug transposon mariner (Genebank Accession No. S37012), and human sequence is the human MARINER (U49974). LINE-1 sequence is derived from line-1 protein open leading frame 2 (B28096). The numbers indicate the amino acid positions in each protein. Multiple stop codons indicated by asterisks implicate HCC-2 as an inactive LINE-1 homologue

**Table 2** Sequence analysis of 100 clones isolated from subtracted HCC cDNA

| Gene | Number of clones |
|---|---|
| HCC-1[a] | 11 |
| Focal adhesion kinase (FAK) | 8 |
| Glutamine synthetase (GS) | 6 |
| M130 | 3 |
| HCC-2[a] | 3 |
| Guanine binding protein iα (Giα) | 2 |
| Deleted in colon cancer (DCC) | 2 |
| Ornithine aminotransferase (OAT) | 2 |
| Miscellaneous | 43 |
| Total | 100 |

[a] Two previously unidentified genes showing no significant homology to genes within the database were designated arbitrarily as HCC-1 and -2.

at the minimal number of PCR cycles for visualization on agarose gels. Amplification of representative house-keeping genes, G3PDH and β-actin, were similar between the HCC and non-HCC tissues. Target products of genes obtained from HCC were clearly amplified from HCC cDNA, whereas only weak or no amplification occurred from non-HCC cDNA at the same PCR cycles. Conversely, decorin was amplified more efficiently from non-HCC tissue than from HCC tissue.

### Quantitative analysis of differentially expressed genes in HCC

The expression levels of mRNA of these 8 known genes were quantitated in 10 pairs of HCC and non-HCC tissues, including the original samples used in SSH. SMART™ cDNAs were generated from each sample, and used as templates of quantitative PCR. The quantity of cDNA copies were standardized with those of G3PDH in each sample. The ratio of each gene in HCC tissue compared to non-HCC tissue is shown in Figure 4. The expression levels of these genes were not always over the detection limits of the PCR assay in all 10 pairs. When the expression level of the target cDNA was under the detection limits in HCC, the ratio was considered to

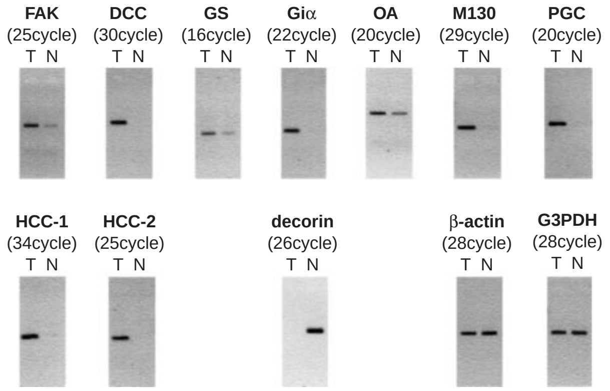

**Figure 3** Confirmation of expression in original HCC by semi-quantitative RT-PCR. RT-PCR using specific primer sets for each subtracted gene were performed with 10 ng of HCC and non-HCC cDNA as a template. Amounts of PCR products reflect gene expressions in the original sample, since PCR products were analysed at the PCR cycle number in the exponential phase of amplification for each gene as indicated below each gene name. Expression of housekeeping genes (β-actin and G3PDH) was at the same levels in HCC and non-HCC tissue. T indicates the tumour tissues, and N indicates non-tumorous tissues

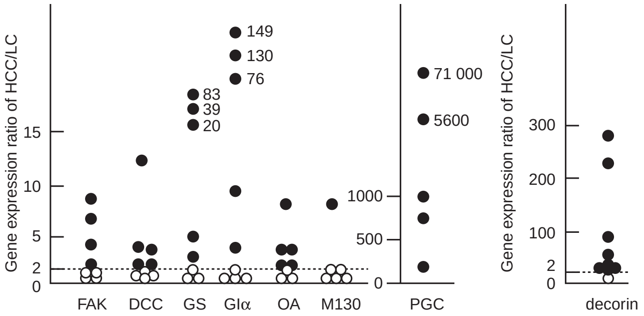

**Figure 4** Gene expression ratio of HCC to non-HCC in paired samples. Expression of each gene was standardized with the expression of G3PDH in each sample. Closed circles represent the cases in which the gene expression ratio of HCC to non-HCC was greater than 2 (except decorin), and open circles less than 2. For decorin, closed circles indicate the cases whose expression ratio of non-HCC/HCC was greater than 2, and open circles correspond to cases whose ratio was less than 2. The numbers beside the circles indicate the ratios in values be 0. When the expression in non-HCC tissue was under the detection limits, the ratio was calculated by dividing the value for HCC by the detection limits of the target gene. The HCC/non-HCC ratio could not be available when the target cDNA could not be quantitated in both HCC and non-HCC tissues of the same patient (2 cases for FAK, 1 case for DCC, 2 case for GS, 1 case for Giα, 2 cases for OAT, 4 cases for M130 and 5 cases for PGC). FAK was overexpressed in HCC compared to non-HCC by more than 2-fold in 4 of 8 cases (2–8.5 folds), DCC in 5 of 9 (2.2–14 folds), GS in 5 of 8 (2–63 folds), Giα in 5 of 9 (8.6–149 folds), OAT in 5 of 8 (2.3–8.3 folds), M130 in 1 of 6 (8.4 folds), PGC was in 5 of 5 cases (5.7–70700 folds). Decorin was suppressed in 8 of 9 cases (2–266 folds). There was no correlation between mRNA levels of the identified genes and the aetiology or the histological grade of tumours.

### Immunohistochemistry

The immunohistochemical analyses for PGC and GS revealed positive staining in hepatoma cells, whereas no apparent signals were detectable in non-HCC tissue (Figure 5). FAK and DCC

*© 2001 Cancer Research Campaign* 

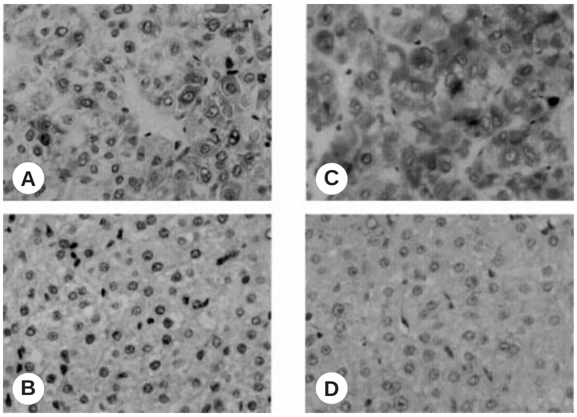

**Figure 5** Immunohistochemistry for PGC and GS in HCC and non-HCC tissue used for SSH analysis. These gene products were specifically stained in hepatoma tissues compared to non-hepatoma tissues. GS in hepatoma cells (**A**). GS in non-hepatoma cells (**B**). PGC in hepatoma cells (**C**). PGC in non-hepatoma cells (**D**)

could not be detected in either HCC or non-HCC tissue (data not shown), probably because their absolute expression levels were too low to be detected by immunostaining.

### DISCUSSION

Although many studies have analysed genes differentially expressed in tumour tissues compared to non-tumorous liver tissue, the comprehensive picture of HCC-specific gene expression has not been revealed. In the present study, we applied the novel subtractive suppression hybridization technique combined with the SMART™ RT-PCR method in order to identify the differentially expressed genes between HCC and non-HCC tissue. As a result, 7 genes were identified as up-regulated and one gene as down-regulated in HCC. In addition, overexpression of 2 previously unknown genes showing weak homology with retrotransposon sequences were also detected. Differential expression of these genes in the index case was demonstrated by the semi-quantitative RT-PCR assay, and increased protein expression of GS and PGC tissue was also confirmed by immunohistochemistry. Quantitative analysis of gene expression using RT-PCR demonstrated the differential expression of these genes in other HCC samples as well as the index sample. Interestingly, most of these genes are associated with HCC or other cancers as described below. Identification of such genes showing altered expression in HCC would be useful step in understanding the molecular pathogenesis of HCC, in developing specific tumor markers, and in designing potentially novel therapeutic directions.

Generally, it is estimated that approximately 20 000 genes are expressed in an individual cell (Zhang et al, 1997), and previous SAGE (Zhang et al, 1997) or high-density microarray (Alon et al, 1999) data have suggested that the proportion of genes whose expression levels in tumours is higher or lower than 10-fold in comparison to normal tissues account for as much as 0.5–2% of mRNA species (Zhang et al, 1997). Such small differences in gene expression between cancer and its origin tissue were also suggested by the present observation that electrophoretic bands of HCC SMART™ cDNA closely resembled that of non-HCC SMART™ cDNA. SSH technique seems to be sensitive enough to detect differentially expressed genes of such low abundance, because in the control experiment that used spiked $\phi$X174 DNA consisting of 0.02% of tester cDNA could be easily recovered by SSH using non-spiked cDNA as a driver. SSH involves a process called normalization, which equalizes the relative amount of each cDNA species in the hybridization step (Diatchenko et al, 1996). This process avoids preferential isolation of genes with high abundance. Thus, differentially expressed genes with low abundance can be detected sensitively, making this procedure more simple and effective than traditional subtraction. In addition, it has been extensively validated by other investigators (Endege et al, 1999) that SMART™ cDNA products can maintain the original message profile from a small amount of RNA.

Out of 7 known genes identified as upregulated in HCC, 2 genes (GS and Gi$\alpha$) were well-established as overexpressed in HCC (Christa et al, 1994, et al, 1997). GS is a ubiquitous enzyme that catalyzes the synthesis of glutamine from glutamate and ammonia at the expense of ATP. Overexpression of GS mRNA in HCC has been reported previously (Christa et al, 1994), and its role in promoting metastasis was suggested (Osada et al, 1999). Gi$\alpha$ is a subunit of guanine-binding inhibitory protein that inhibits adenylate cyclase and is known to be involved in cell growth, differentiation, apoptosis. 5 other known genes detected in this study were not previously reported as being up-regulated in HCC.

PGC is a proteolytic enzyme that digests proteins in the stomach and is also involved in gastric epithelial cell growth during gastric mucosal healing (Kishi et al, 1997). It is produced not only in stomach, but also in breast cancer (Diez-Itza et al, 1993) and prostate cancer (Konishi et al, 1999), and is implicated in the lytic processes of invasive cancer lesions. Extragastric expression of PGC is partially regulated with a minimal promoter region that is found in androgen-regulated genes (Balbin and Lopez-Otin, 1996). Actually, in breast cancer and prostate cancer, expression of PGC is closely associated with androgen receptor status (Diez-Itza et al, 1993; Konishi et al, 1999), indicating that PGC expression is regulated by androgens in these tumours. HCC is a male predominant disease and expresses the androgen receptor (Nagasue et al, 1995), suggesting that the expression of PGC in HCC might be also up-regulated by androgens. Although the role of PGC in HCC is still unknown, the common pathway of tumour growth or tumorigenesis between HCC and other PGC-producing cancers may be found.

FAK is a signal transducer of integrins and some soluble growth factors. FAK becomes phosphorylated and activated during integrin-mediated cell adhesion, of which signal allows cells to sense adherence to the extracellular matrix, thus providing a cell survival signal and preventing apoptosis (Frisch et al, 1996). Cells which express FAK show increased migration relative to wild-type cells (Kornberg, 1998). FAK has been reported to overexpress in invasive and metastatic colon (Weiner et al, 1993), thyroid (Owens et al, 1996) and prostate cancers (Tremblay et al, 1996), but its overexpression in mRNA or protein level has never been reported in HCC. Although mechanisms of up-regulation of FAK is unknown, overexpression of FAK leads to increased cell migration and increased cell survival under anchorage-independent conditions (Weiner et al, 1993), which might be advantageous to HCC growth and invasion. Further work is needed to determine whether FAK represents a therapeutic target for invasive HCC. We found no mutations via sequencing analysis of the full length of FAK as well as PGC cDNA obtained from the original HCC sample, suggesting as for the index case that overexpression of these genes was not a compensatory mechanism for inactivating mutations.

 © 2001 Cancer Research Campaign

The DCC gene codes a protein with significant sequence similarity to neural cell adhesion molecules, a transmembrane protein of the immunoglobulin superfamily, which is thought to be a receptor for netrin-1 (Fazeli et al, 1997). DCC induces apoptosis in the absence of netrin-1 leading to the inhibition of metastasis or invasion beyond local blood supply (Mehlen et al, 1998). While the DCC gene was expressed in normal colonic mucosa or non-metastatic colon cancers, its expression was greatly reduced in invasive or metastatic adenocarcinomas with unfavourable prognoses (Gotley et al, 1996). In normal tissues, DCC protein is abundant in bladder tissue, and is detectable in colon, pancreas, and kidney, but not in normal liver tissue (Gotley et al, 1996). Therefore, retained expression of DCC in HCC found in this study might prevent hepatoma cells from invasion and metastasis. In fact, the index case was obtained from a primary lesion with no distant metastasis (Mehlen et al, 1998). DCC expression within metastatic hepatomas should be examined in order to elucidate the preventive role of DCC in HCC progression.

The relationship between hepatocarcinogensis and the other 2 detected genes, OAT and M130 is rather unclear. OAT catalyses the transamination of ornithine to glutamate, substrate of GS in the liver (Kuo and Darnell, 1991). M130 is a macrophage differentiation antigen which is a member of the scavenger receptor superfamily (Ritter et al, 1999) and differential expression in mesothelial cancer was reported (Frank et al, 1998).

On the contrary, SSH analysis of non-HCC minus HCC identified decorin as being down-regulated in HCC, which was suppressed in 8 of 9 HCC tissues examined. Decorin is a small proteoglycan, and is known to inhibit transforming growth facter-β (TGF-β) by binding to it, and may directly interfere with the cell cycle via induction of cyclin-dependent protein kinase, p21 (Stander et al, 1999). It has been reported that decorin suppresses tumorigenicity when expressed in colon cancer cells (Santra et al, 1995), but the association with HCC has not yet been elucidated. The present results suggest that decorin might have an inhibitory effect on HCC tumorigenesis or progression. Further studies on the role of decorin in hepatocarcinogenesis are warranted.

Two previously unknown genes, HCC-1 and HCC-2, were found to be overexpressed in HCC and showed weak homologies to the retrotransposon sequences, MARINER (Robertson et al, 1996; Plasterk et al, 1999) and LINE-1 (Skowronski et al, 1988; Bratthauer and Fanning, 1992), respectively. Although in murine hepatoma models both carcinogen-induced and spontaneous liver tumour formation is associated with abnormalities in the expression of endogenous retrovirus-related DNA sequences (Dragani et al, 1986), the actual function of HCC-1 and HCC-2 in HCC is unknown and awaits the further characterization.

Since the case analysed by SSH was moderately differentiated carcinoma, the detected genes might be overexpressed specifically in moderately differentiated HCC. HCC of other differentiation grade tumours may possess different expression profiles from the one obtained in this study, thus different stages of HCC should be analysed to elucidate the complete picture of the stage-specific gene expressions.

In conclusion, SSH showed that PGC and FAK are overexpressed and decorin is suppressed in HCC, demonstrating that it is applicable for revealing the previously unknown, differential gene expression from a small clinical samples. The information of such alterations in gene expression could be useful for elucidating the genetic events in HCC pathogenesis and developing the new diagnosic marker or therapeutic targets.

### ACKNOWLEDGEMENTS

Wc thank Dr Naoya Sakamoto, Dr Masayuki Kurosaki, Dr Shinhan Yu, the Second Department of Internal Medicine, Tokyo Medical and Dental University for critical discussion of the study, and also Dr Tsuneo Natori, Department of Inter-Laboratory Test Management, SRL, Hachioji, Tokyo for technical assistance in immunohistochemistry. A part of this study was supported by the Program for Promotion of Fundamental Studies in Health Sciences of the Organization for Drug ADR Relief, R&D Promotion and Product Review of Japan, and Grant in Aid (08457164) by the Ministry of Education, Science, Culture and Sports of Japan.

### REFERENCES

Alon U, Barkai N, Notterman DA, Gish K, Ybarra S, Mack D and Levine AJ (1999) Broad patterns of gene expression revealed by clustering analysis of tumor and normal colon tissues probed by oligonucleotide arrays. *Proc Natl Acad Sci USA* **96**: 6745–6750

Balbin M and Lopez-Otin C (1996) Hormonal regulation of the human pepsinogen C gene in breast cancer cells. Identification of a cis-acting element mediating its induction by androgens, glucocorticoids, and progesterone. *J Biol Chem* **271**: 15175–15181

Bratthauer GL and Fanning TG (1992) Active LINE-1 retrotransposons in human testicular cancer. *Oncogene* **7**: 507–510

Chomczynski P, Sacchi N, Siebert PD, Lukyanov SA, Sverdlov ED and Berg DE (1987) Single-step method of RNA isolation by acid guanidinium thiocyanate-phenol-chloroform extraction. *Anal Biochem* **162**: 156–159

Christa L, Simon MT, Flinois JP, Gebhardt R, Brechot C and Lasserre C (1994) Overexpression of glutamine synthetase in human primary liver cancer. *Gastroenterology* **106**: 1312–1320

Diatchenko L, Lau YF, Campbell AP, Chenchik A, Moqadam F, Huang B, Lukyanov S, Lukyanov K, Gurskaya N, Sverdlov ED and Siebert PD (1996) Suppression subtractive hybridization: a method for generating differentially regulated or tissue-specific cDNA probes and libraries. *Proc Natl Acad Sci USA* **93**: 6025–6030

Diez-Itza I, Merino AM, Tolivia J, Vizoso F, Sanchez LM and Lopez-Otin C (1993) Expression of pepsinogen C in human breast tumours and correlation with clinicopathologic parameters. *Br J Cancer* **68**: 637–640

Dragani TA, Manenti G, Della Porta G, Gattoni-Celli S and Weinstein IB (1986) Expression of retroviral sequences and oncogenes in murine hepatocellular tumors. *Cancer Res* **46**: 1915–1919

el-Deiry WS, Tokino T, Velculescu VE, Levy DB, Parsons R, Trent JM, Lin D, Mercer WE, Kinzler KW and Vogelstein B (1993) WAF1, a potential mediator of p53 tumor suppression. *Cell* **75**: 817–825

Endege WO, Steinmann KE, Boardman LA, Thibodeau SN and Schlegel R (1999) Representative cDNA libraries and their utility in gene expression profiling. *Biotechniques* **26**: 542–548, 550

Fazeli A, Dickinson SL, Hermiston ML, Tighe RV, Steen RG, Small CG, Stoeckli ET, Keino-Masu K, Masu M, Rayburn H, Simons J, Bronson RT, Gordon JI, Tessier-Lavigne M and Weinberg RA (1997) Phenotype of mice lacking functional Deleted in colorectal cancer (DCC) gene. *Nature* **386**: 796–804

Frank S, von Specht BU, Farthmann EH and Hirsch T (1998) Identification of genes involved in human mesothelial cancer progression using a modified differential display technique. *Cancer Lett* **123**: 7–14

Frisch SM, Vuori K, Ruoslahti E and Chan-Hui PY (1996) Control of adhesion-dependent cell survival by focal adhesion kinase. *J Cell Biol* **134**: 793–799

Gotley DC, Reeder JA, Fawcett J, Walsh MD, Bates P, Simmons DL and Antalis TM (1996) The deleted in colon cancer (DCC) gene is consistently expressed in colorectal cancers and metastases. *Oncogene* **13**: 787–795

Khan J, Simon R, Bittner M, Chen Y, Leighton SB, Pohida T, Smith PD, Jiang Y, Gooden GC, Trent JM and Meltzer PS (1998) Gene expression profiling of alveolar rhabdomyosarcoma with cDNA microarrays. *Cancer Res* **58**: 5009–5013

Kishi K, Kinoshita Y, Matsushima Y, Okada A, Maekawa T, Kawanami C, Watanabe N and Chiba T (1997) Pepsinogen C gene product is a possible growth factor during gastric mucosal healing. *Biochem Biophys Res Commun* **238**: 17–20

Konishi N, Nakaoka S, Matsumoto K, Nakamura M, Kuwashima S, Hiasa Y, Cho M, Uemura H and Hirao Y (1999) Expression of pepsinogen II with androgen and estrogen receptors in human prostate carcinoma. *Pathol Int* **49**: 203–207

© 2001 Cancer Research Campaign 

Kornberg LJ (1998) Focal adhesion kinase and its potential involvement in tumor invasion and metastasis. *Head Neck* **20**: 745–752

Kuang WW, Thompson DA, Hoch RV, Weigel RJ, Siebert PD, Lukyanov SA, Sverdlov ED and Berg DE (1998) Differential screening and suppression subtractive hybridization identified genes differentially expressed in an estrogen receptor-positive breast carcinoma cell line. *Nucleic Acids Res* **26**: 1116–1123

Liang P, Averboukh L, Keyomarsi K, Sager R and Pardee AB (1992) Differential display and cloning of messenger RNAs from human breast cancer versus mammary epithelial cells. *Cancer Res* **52**: 6966–6968

Matz M, Shagin D, Bogdanova E, Britanova O, Lukyanov S, Diatchenko L and Chenchik A (1999) Amplification of cDNA ends based on template-switching effect and step-out PCR. *Nucleic Acids Res* **27**: 1558–1560

Mehlen P, Rabizadeh S, Snipas SJ, Assa-Munt N, Salvesen GS and Bredesen DE (1998) The DCC gene product induces apoptosis by a mechanism requiring receptor proteolysis. *Nature* **395**: 801–804

Nagasue N, Yu L, Yukaya H, Kohno H and Nakamura T (1995) Androgen and oestrogen receptors in hepatocellular carcinoma and surrounding liver parenchyma: impact on intrahepatic recurrence after hepatic resection. *Br J Surg* **82**: 542–547

Osada T, Sakamoto M, Nagawa H, Yamamoto J, Matsuno Y, Iwamatsu A, Muto T and Hirohashi S (1999) Acquisition of glutamine synthetase expression in human hepatocarcinogenesis: relation to disease recurrence and possible regulation by ubiquitin-dependent proteolysis. *Cancer* **85**: 819–831

Owens LV, Xu L, Dent GA, Yang X, Sturge GC, Craven RJ and Cance WG (1996) Focal adhesion kinase as a marker of invasive potential in differentiated human thyroid cancer. *Ann Surg Oncol* **3**: 100–105

Plasterk RH, Izsvak Z and Ivics Z (1999) Resident aliens: the Tc1/mariner superfamily of transposable elements. *Trends Genet* **15**: 326–332

Ritter M, Buechler C, Langmann T and Schmitz G (1999) Genomic organization and chromosomal localization of the human CD163 (M130) gene: a member of the scavenger receptor cysteine-rich superfamily. *Biochem Biophys Res Commun* **260**: 466–474

Robertson HM, Zumpano KL, Lohe AR and Hartl DL (1996) Reconstructing the ancient mariners of humans. *Nat Genet* **12**: 360–361

Santra M, Skorski T, Calabretta B, Lattime EC and Iozzo RV (1995)De novo decorin gene expression suppresses the malignant phenotype in human colon cancer cells. *Proc Natl Acad Sci USA* **92**: 7016–7020

Sherlock S and Dooley J (1997) *Disease of the liver and biliary system* Blackwell Science: Oxford.

Skowronski J, Fanning TG and Singer MF (1988) Unit-length line-1 transcripts in human teratocarcinoma cells. *Mol Cell Biol* **8**: 1385–1397

Stander M, Naumann U, Wick W and Weller M (1999) Transforming growth factor-beta and p-21: multiple molecular targets of decorin-mediated suppression of neoplastic growth. *Cell Tissue Res* **296**: 221–227

Stubbs AP, Abel PD, Golding M, Bhangal G, Wang Q, Waxman J, Stamp GW and Lalani EN (1999) Differentially expressed genes in hormone refractory prostate cancer: association with chromosomal regions involved with genetic aberrations. *Am J Pathol* **154**: 1335–1343

Tremblay L, Hauck W, Aprikian AG, Begin LR, Chapdelaine A and Chevalier S (1996) Focal adhesion kinase (pp125FAK) expression, activation and association with paxillin and p50CSK in human metastatic prostate carcinoma. *Int J Cancer* **68**: 164–171

Vasmatzis G, Essand M, Brinkmann U, Lee B and Pastan I (1998) Discovery of three genes specifically expressed in human prostate by expressed sequence tag database analysis. *Proc Natl Acad Sci USA* **95**: 300–304

Velculescu VE, Zhang L, Vogelstein B and Kinzler KW (1995) Serial analysis of gene expression. *Science* **270**: 484–487

von Stein OD, Thies WG and Hofmann M (1997) A high throughput screening for rarely transcribed differentially expressed genes. *Nucleic Acids Res* **25**: 2598–2602

Weiner TM, Liu ET, Craven RJ and Cance WG (1993) Expression of focal adhesion kinase gene and invasive cancer. *Lancet* **342**: 1024–1025

Wittwer CT, Ririe KM, Andrew RV, David DA, Gundry RA, Balis UJ, Siebert PD, Lukyanov SA, Sverdlov ED and Berg DE (1997) The LightCycler: a microvolume multisample fluorimeter with rapid temperature control. *Biotechniques* **22**: 176–181

Zachar V, Thomas RA, Goustin AS, Siebert PD, Lukyanov SA, Sverdlov ED and Berg DE (1993) Absolute quantification of target DNA: a simple competitive PCR for efficient analysis of multiple samples. *Nucleic Acids Res* **21**: 2017–2018

Zhang L, Zhou W, Velculescu VE, Kern SE, Hruban RH, Hamilton SR, Vogelstein B and Kinzler KW (1997) Gene expression profiles in normal and cancer cells. *Science* **276**: 1268–1272

*© 2001 Cancer Research Campaign*
